# Larvicidal Activity of Phytochemicals From *Handroanthus impetiginosus* Seeds Against *Culex quinquefasciatus*

**DOI:** 10.1002/cbdv.202501126

**Published:** 2025-07-31

**Authors:** Luana Cristina Diniz Santos, Lucas Santos Azevedo, Ezequias Pessoa de Siqueira, Mairon César Coimbra, Stênio Nunes Alves, Ana Hortência Fonsêca Castro, Luciana Alves Rodrigues dos Santos Lima

**Affiliations:** ^1^ Universidade Federal de São João Del‐Rei Divinópolis Minas Gerais Brazil; ^2^ Centro De Pesquisa René Rachou—Fiocruz/MG Belo Horizonte Minas Gerais Brazil

**Keywords:** bioactive substances | fatty acids | *Handroanthus* | inseticidal activity | toxicity

## Abstract

*Handroanthus impetiginosus* is a species known to produce bioactive substances, including compounds with larvicidal activity. This study evaluated the toxicity of hexane extract (HE), fatty acids (FAs), and FA methyl esters (FAMEs) from *H. impetiginosu*s seeds against *Culex quinquefasciatus* larvae. FAs were obtained by hydrolysis of triglycerides present in HE for 30 and 60 min, and FAMEs were produced by transesterification of FAs for the same time. HE was analyzed by gas chromatography–mass spectrometry (GC–MS). Oleic acid and ethyl oleate were found in abundance in HE. HE, FA30s, and FA60s produced mortality rates above 90% in *C. quinquefasciatus* larvae; FA60s produced a 100% mortality rate at the highest concentration at which they were tested (250 µg/mL). LD_50_ values ranged from 59.70 µg/mL for HE to 1255.41 µg/mL for FAME60s. LD_90_ ranged from 194.72 µg/mL for FA30s to 295.56 µg/mL for FA60s. The results indicate that HE and FAs have larvicidal potential.

## Graphical abstract

The hexane extract was obtained from the seeds of *H. impetiginosus*, from which fatty acids and FAMEs were obtained. The three samples were tested on *C. quinquefasciatus* larvae, promoting mortality with moderate LC_50_ values. The hexane extract was analyzed by gas chromatographymass spectrometry (GC‐MS), and the most abundant compounds were ethyl oleate (31.52%), ethyl linoleate (18.97%), ethyl palmitate (15.42%), ethyl stearate (8.82%), and oleic acid (13.22%). Above is the *H. impetiginosus* tree, below are the seeds of the species. GC‐MS and brackets show the most abundant compounds in the hexane extract, and to the side, the chromatogram obtained. The figure above and in the center shows a larva of the mosquito *C. quinquefasciatus*.

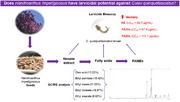

## 1 Introduction

Mosquitoes are significant vectors of several human diseases [1]. Globally, around 700 million people are infected annually through mosquito‐borne pathogens, with over one million fatalities reported each year [2]. These insects, belonging to the order Diptera, suborder Nematocera, and family Culicidae [3], play a central role in the epidemiology of various diseases. *Culex quinquefasciatus*, in particular, is a vector of arboviruses including Saint Louis encephalitis, Eastern and Western equine encephalitis, Venezuelan equine encephalitis, and West Nile virus [4], as well as *Wuchereria bancrofti*, the etiological agent of lymphatic filariasis [5].

Although synthetic insecticides are still widely used in urban mosquito control, their long‐term application has led to the development of resistance in vector populations, environmental toxicity, and health risks due to improper use [6, 7]. These limitations underscore the urgent need for environmentally sound and sustainable alternatives. Plants are an excellent source for the development of natural insecticides, as plant formulations and their constituents can meet commercial criteria and pose a lower health risk. In addition, secondary metabolites can act on new target sites and thus reduce the resistance to synthetic insecticides [8].

Over the past two decades, more than 2000 plant species have been identified as sources of bioactive compounds with larvicidal potential against mosquitoes [9, 10, 11]. Plant seed oils are rich in fatty acids (FAs), and the esterification reaction of FAs produces methyl esters (FAMEs). These compounds have shown larvicidal action on mosquitoes, and the use of these molecules in insect control has increased [12, 13]. Some mechanisms, such as cell death, are reported to be involved in the insecticidal action of plant bioactive compounds. These molecules are capable of provoking or disturbing insect physiology in different ways, affecting the nervous system, degrading the cell membrane, or promoting complete metamorphosis of the insect [14].

Notably, plant extracts have also demonstrated efficacy against other arthropod pests such as mites, as illustrated by Hemmat‐Jou et al., who reported the acaricidal potential of *Euphorbia* species against *Tetranychus urticae* [15]. These findings highlight the broader pest control potential of phytochemicals and justify continued research for novel botanical sources.

*Handroanthus impetiginosus* (Mart. ex DC.) Mattos (Bignoniaceae), which has as synonyms *Tabebuia impetiginosa* (Martius ex DC.) Standl., *Tabebuia avellanedae* Lorentz ex Griseb., *Handroanthus avellanedae* (Lorentz ex Griseb.) Mattos, is a species from the seasonal forest used in folk medicine to treat infections, fevers, ulcers, and cancer. This species is rich in bioactive compounds such as naphthoquinones, coumarins, flavonoids, iridoids, and benzoic acid derivatives found mainly in the stem bark [16, 17, 18]. Its biological properties include antimicrobial, antioxidant, anti‐inflammatory, and antineoplastic activities [18, 19].

The current study focuses on seeds, a less explored organ of the plant, and evaluates the activity of their lipophilic constituents against *C. quinquefasciatus* larvae. A previous study demonstrated that the hexane extract (HE), FAs, and FAMEs from *H. impetiginosus* seeds showed significant antioxidant potential and toxicity against *Artemia salina* in lethality bioassay [20]. Hexane was selected as the extraction solvent due to its nonpolar nature, which facilitates the isolation of FAs and esters, compounds previously reported as effective larvicides and acaricides [10, 11, 21, 22].

Despite growing evidence supporting the use of plant‐based larvicides, limited attention has been given to seed‐derived nonpolar extracts from *H. impetiginosus* and their potential for mosquito control. This study aimed to assess the larvicidal activity of the HE, FAMEs, and FAs from *H. impetiginosus* seeds against *C. quinquefasciatus*. This study is expected to contribute to enriching the database of phytochemicals used in mosquito control and reinforce the importance of using sustainable biodiversity‐based strategies for vector control.

## 2 Results and Discussion

### 2.1 GC/MS Analysis of HE

The HE was analyzed by gas chromatography–mass spectrometry (GC–MS) (see Supporting Information) to characterize its chemical constituents (Table 1). The majority of the components present in the seeds were FAs and ethyl esters: The most abundant compounds were ethyl oleate (31.52%), ethyl linoleate (18.97%), ethyl palmitate (15.42%), ethyl stearate (8.82%), and oleic acid (13.22%).

**TABLE 1:** Composition and percentages of compounds in the hexane extract (HE) from seeds of *Handroanthus impetiginosus*.

RT (min)	Compounds	RI	RIL	%	Mass spectrometry data	Molecular formula
26.311	Palmitic acid	1964	1960(a)	2.78	256	C_16_H_32_O_2_
26.785	Ethyl palmitate	1997	1993(b)	15.42	284	C_18_H_36_O_2_
28.320	Methyl oleate	2106	2095(a)	0.44	296	C_19_H_36_O_2_
28.858	Oleic acid	2146	2142(a)	13.22	282	C_18_H_34_O_2_
29.159	Ethyl linoleate	2168	2151(b)	18.97	308	C_20_H_36_O_2_
29.225	Ethyl oleate	2173	2173(b)	31.52	310	C_20_H_38_O_2_
29.545	Ethyl stearate	2197	2196(a)	8.82	312	C_20_H_40_O_2_
31.551	NI	2355	—	0.56	—	—
31.729	NI	2369	—	1.03	—	—
31.790	NI	2374	—	0.24	—	—
32.081	Ethyl eicosanoate	2398	2390(a)	1.49	340	C_22_H_44_O_2_
34.423	Ethyl docosanoate	2599	2593(b)	0.45	368	C_24_H_48_O_2_
36.750	Ethyl tetracosanoate	2799	2779(b)	0.32	396	C_26_H_52_O_2_
37.289	Squalene	2838	2835(b)	0.45	410	C_30_H_50_
39.204	NI	2964	—	4.30	—	—

*Note*: RI: retention index of compound on RTx‐5MS; RIL: retention index of literature on DB‐5MS (a) and HP‐5MS (b).

Abbreviation: RT: retention time.

Oleic acid, palmitic acid, and linoleic acid had already been identified in the seeds and bark of *H. impetiginosus* in previous studies [18, 23, 24]. Oleic acid has also been identified as the most abundant FA found in the seed oil of this species [24]. However, to the best of our knowledge, our current study is the first to identify ethyl esters in *H. impetiginosus*.

### 2.2 Fourier Transform Infrared (FTIR) Analysis of HE

**FIGURE 1:**
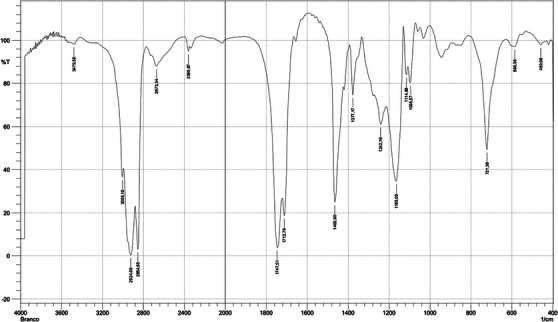
FTIR spectral peaks of hexane extract (HE) of *Handroanthus impetiginosus*.

The presence of functional groups in the HE was analyzed through FTIR Spectroscopy (Figure 1). The stronger bands occurred at 2924.09 and 2854.65 cm^−1^ (associated to the stretching vibration from C─H), 1747.51 and 1712.79 cm^−1^ (related to the C═O stretching vibration from carbonyl groups), 1465.9 cm^−1^ (associated with bending vibrations of C─H), 1377.17 cm^−1^ (associated with ester C─O stretching), 1242.16 cm^−1^ (related to C(═O)─O stretching vibrations of ester), 1165 cm^−1^ (associated from O─C─C stretching vibrations), and 721.38 cm^−1^ (related with CH_2_ rocking vibrations). This information confirms the characterization carried out by the GC/MS analysis.

### 2.3 Larvicidal Activity on *Culex quinquefasciatus* Larvae

Table 2 shows the mortality rates of *C. quinquefasciatus* larvae after exposure to samples (HE, FA30s, FA60s, FAME30s, and FAME60s) at various concentrations. The results demonstrated a dose‐dependent relationship between the concentrations used and larval mortality rates. The HE, FA30s, and FA60s each produced mortality rates above 90% in *C. quinquefasciatus* larvae; a 100% mortality rate was observed when FA60s was used at its highest tested concentration (250 µg/mL), followed by HE and FA30s, with 96.67% and 91.67% mortality rates, respectively (*p* < 0.05), at the same concentration. The mortality rate remained high when HE (73.33%) and FA30s (83.33%) were tested at 125 µg/mL and remained above 50% when HE was tested at 62.5 µg/mL (*p* < 0.05). Tests using 31.25 µg/mL yielded mortality rates below 20%. These results demonstrate the larvicidal activities of the tested samples, especially HE and FA30s, and indicate a possible insecticidal activity of *H. impetiginosus* extract and its derivatives.

**TABLE 2:** Larvicidal activity of different concentrations of the extract, fatty acids, and methyl esters from seeds of *Handroanthus impetiginosus* on *Culex quinquefasciatus* larvae.

Samples	% Mortality					LC_50_ (µg/mL)	LC_90_ (µg/mL)
	15.62 µg/mL	31.25 µg/mL	62.5 µg/mL	125 µg/mL	250 µg/mL		
HE	11.67 ± 1.67*	23.33 ± 1.67*	53.33 ± 7.26*	73.33 ± 1.67*	96.67 ± 1.67*	59.70 (47.43–75.15)	288.05 (145.63–357.10)
FA30s	10.0*	15.00 ± 2.89*	43.33 ± 1.67*	83.33 ± 3.33*	91.67 ± 1.67*	67.40 (56.20–80.84)	194.72 (159.41–237.86)
FAME30s	5.00	11.67 ± 1.67*	13.33 ± 1.67*	26.67 ± 3.33*	48.33 ± 8.33*	268.30 (194.37–370.35)	ND
FA60s	3.33 ± 1.67	13.33 ± 3.33*	30.00 ± 2.89*	40.00 ± 5.00*	100.00*	111.10 (87.51–141.05)	295.56 (226.88–385.02)
FAME60s	5.00	10.00*	16.67 ± 1.67*	21.67 ± 1.67*	26.67 ± 1.67*	1255.41 (573.55–1747.90)	ND
Control	0	0	0	0	0	ND	ND

*Note*: FA30s: fatty acids of HE obtained by hydrolysis for 30 min, FAME30s: fatty acids methyl esters obtained by hydrolysis of fatty acids for 30 min, FA60s: fatty acids of HE obtained by hydrolysis for 60 min, FAME60s: fatty acids methyl esters obtained by hydrolysis of fatty acids for 60 min. LC_50_: lethal concentration for 50% of the population. LC_90_: lethal concentration for 90% of the population. The results are means ± SE (*n* = 3). *p < 0.05 for compared to control by Tukey test.

Abbreviations: HE, hexane extract; ND, not determined.

The LC_50_ and LC_90_ values for HE, FA30s, FA60s, FAME30s, and FAME60s are shown in Table 2. HE exhibited the lowest LC_50_ (59.70 µg/mL) and an LC_90_ of 288.05 µg/mL, whereas FA30s had an LC_50_ of 67.40 µg/mL and an LC_90_ of 194.72 µg/mL. FA60s showed LC_50_ and LC_90_ values of 111.10 and 295.56 µg/mL, respectively, and FAME30s gave an LC_50_ of 268.30 µg/mL; it was not possible to calculate LC values for FAME60s.

According to Komalamisra et al. [25], compounds with LC_50_ > 750 µg/mL are non‐toxic, LC_50_ between 100 and 750 µg/mL are not very effective, LC_50_ between 50 and 100 µg/mL are moderately effective, and LC_50_ ≤ 50 µg/mL are effective. Thus, HE and FA30s showed moderate activity, whereas FA60s and FAME30s showed low activity.

No reports were found in the literature on the larvicidal activity of *H. impetiginosus* on *C. quinquefasciatus*. However, Kim et al. [26] demonstrated the larvicidal activity of *T. avellanedae* bark extracts on *Aedes aegypti*, *Culex pipiens pallens*, and *Ochlerotatus togoi*. The methanol extract killed 100% of larvae of all three mosquito species at 50–100 µg/mL, and the chloroform extract achieved 100% mortality at 100 µg/mL. Borges et al. [27] evaluated acetone and ethanol extracts of *T. avellanedae* wood against *A. aegypti* larvae, reporting LC_50_ values of 1.499 and 4.633 µg/mL, respectively. These extracts also strongly inhibited oviposition at 333.3 µg/mL.

The toxicity of FAs and FAMEs has been previously evaluated in mosquito larvae. A study assessed the larvicidal activity of FAs from vegetable oils and their respective methyl esters in *C. quinquefasciatus* larvae, which presented LC_50_ values between 42.32 and 286.33 µg/mL, with lower rates for FAMEs [28]. De Melo et al. [12] tested oleic, linoleic, and linolenic acids on *C. quinquefasciatus*, with LC_50_ values of 8.58, 10.04, and 19.78 µg/mL, respectively. In this study, unsaturated FAs demonstrated selectivity on *C. quinquefasciatus* larvae, because saturated FAs (palmitic and stearic) were not very active. Silva et al. [29] tested oils, FAs, and FAMEs from unripe and ripe fruits of *Solanum lycocarpum*, showing that FAs were most lethal, with palmitic, stearic, oleic, and linoleic acids as major components. Azevedo et al. [30] demonstrated the larvicidal potential of ether extracts, FAs, and FAMEs from *Tecoma stans* seeds against *C. quinquefasciatus*, identifying palmitic, stearic, and oleic acids as key contributors, with an LC_50_ of 20.06 µg/mL.

Ethyl linoleate, isolated from the ethanol extract of *Veratrum lobelianum* rhizomes, was effective against *A. aegypti*, with LC_50_ and LC_90_ values of 24.1 and 38.2 µg/mL, respectively, after 24 h [31]. Sugauara et al. [32] reported that the ethanol extract of *Brunfelsia uniflora* leaves showed larvicidal activity against *A. aegypti*, with ethyl palmitate among the main components (LC_50_ = 4.89 mg/mL). Wang et al. [33] identified ethyl oleate as a pheromone component produced in the thoracic, abdominal, and wing tissues of sexually mature adult and non‐pregnant of *A. aegypti* and detected it in mosquito antennae, suggesting action via sensory receptors and a role in mosquito communication.

In our study, oleic acid was found in the HE, and palmitic, stearic, oleic, and linoleic acids were identified in FA30s and FA60s, with oleic acid as the most abundant compound [20]. Ethyl oleate, ethyl linoleate, and ethyl palmitate were also identified in HE. Thus, the larvicidal activity of HE, FA30s, and FA60s can be attributed, at least in part, to FAs and ethyl esters, supporting previous findings.

De Melo et al. [12] showed metabolic changes in glucose, triglycerides, and protein levels in *C. quinquefasciatus* larvae exposed to the LC_50_ of unsaturated acids (oleic, linoleic, and linolenic acids), resulting in insect stress, which can lead to death. Perumalsamy et al. [8] evaluated the larvicidal activity of FAs and methyl esters on *A. aegypti*, *Aedes albopictus*, and *C. pipiens pallens*. The study demonstrates the structure–activity relationship and suggests that the degree of saturation, side chain length, and geometric isomerism of FAs play a role in determining their toxicity. For example, oleic and palmitic acids act on acetylcholinesterase (AChE), whereas linoleic and linolenic acids may act on AChE and also on the octopaminergic receptor [8]. The main function of AChE is to hydrolyze acetylcholine into choline and acetic acid. Inhibition of AChE increases acetylcholine levels in synapses and causes muscle cramps followed by paralysis and insect death [34, 35]. The biogenic amine octopamine is involved in the control of several physiological and behavioral processes, and together with its receptor, it participates in reproduction, odor perception, metabolism, and homeostasis in insects [36]. Interference in the octopaminergic receptor can alter physiological processes in the mosquito, leading to death.

HE was not toxic to *A. salina*, showing no nauplii mortality even at 1000 µg/mL [20]. FA30s and FA60s presented LC_50_ values of 599.89 and 694.21 µg/mL, respectively, on *A. salina* (Table 3), values higher than those for *C. quinquefasciatus*, with a selectivity index (SI) of 8.9 and 6.3, respectively. Furthermore, at 250 µg/mL, FA30s and FA60s did not induce mortality in *A. salina* (data not shown). These results suggest selectivity of HE, FA30s, and FA60s toward *C. quinquefasciatus*, reinforcing their potential as insecticidal agents.

**TABLE 3:** LC_50_ values of the extract, fatty acids and methyl esters from seeds of *Handroanthus impetiginosus* on *Culex quinquefasciatus* larvae and *Artemia salina*, and selectivity index (SI).

Samples	*Culex quinquefasciatus* LC_50_ (µg/mL)	*Artemia salina* LC_50_ (µg/mL)	SI
**HE**	59.70	>1000^a^	>16
**FA30s**	67.40	599.89	8.9
**FAME30s**	268.30	3.78^a^	0.014
**FA60s**	111.10	694.21	6.30
**FAME60s**	1255.41	24.04^a^	0.019

*Note*: FA30s: fatty acids of HE obtained by hydrolysis for 30 min, FAME30s: fatty acids methyl esters obtained by hydrolysis of fatty acids for 30 min, FA60s: fatty acids of HE obtained by hydrolysis for 60 min, FAME60s: fatty acids methyl esters obtained by hydrolysis of fatty acids for 60 min. LC_50_: lethal concentration for 50% of the population. SI (selectivity index) calculated as the ratio LC_50_ on *A. salina* and LC_50_ on *C. quinquefasciatus*.

^a^ Data obtained from Santos et al. [20].

In addition, recent studies by Hemmat‐Jou et al. [15] provided evidence for the acaricidal potential of plant‐derived metabolites, suggesting that these compounds may exert toxicity by disrupting metabolic, enzymatic, or neuroendocrine pathways of arthropods. Such effects may include interference with mitochondrial respiration, oxidative stress induction, inhibition of AChE activity, or impairment of molting and hormonal regulation. This highlights the importance of further exploring the physiological mechanisms behind the larvicidal actions of FAs and esters present in *H. impetiginosus* extracts.

## 3 Conclusions

Ethyl oleate, ethyl linoleate, ethyl palmitate, ethyl stearate, ethyl eicosanoate, ethyl docosanoate, and ethyl tetracosanoate were detected in *H. impetiginosus* for the first time in this study, to the best of our knowledge. The HE and FAs that were tested showed great larvicidal potential, producing high mortality rates in *C. quinquefasciatus* larvae at the highest concentrations tested. The results from this study suggest that larvicidal activity can be attributed, at least in part, to the presence of FAs and ethyl esters. These results encourage future studies to assess the usefulness of the HE and FAs of *H. impetiginosus* seeds as bioinsecticides.

## 4 Experimental Section

### 4.1 Chemicals

Hexane, hydrochloric acid, potassium hydroxide, and sulfuric acid were purchased from Vetec (Brazil). Dimethylsulfoxide (DMSO) was obtained from Sigma (St. Louis, MO, USA).

### 4.2 Plant Materials

*H. impetiginosus* seeds were collected in Ijaci, Minas Gerais State, Brazil (21°09′97″ S and 44°55′65″ W GRW, at 835 m altitude) (SISBIO no. 24542‐3) between September and October 2019. The vouchers were identified by Andréia Fonseca Silva and deposited in the PAMG Herbarium (PAMG 57021) at the Agricultural Research Company of Minas Gerais (EPAMIG). This study has access permission to the components of genetic heritage, and it is registered in the SisGen Platform (Register A21AFD9), according to Brazilian Biodiversity Law (13.123/2015).

### 4.3 Extraction

HE, FAs (FA30s and FA60s), and FA methyl esters (FAME30s and FAME60s) were obtained according to Santos et al. [20]. After removing the solvent, 134.040 g of HE, 2.248 g of FA30s, 1.972 g of FA60s, 1.683 g of FAME30s, and 1.798 g of FAME60s were obtained.

### 4.4 GC/MS Analysis of HE

GC–MS was used for analysis of extract to characterize its constituents. The equipment Shimadzu GCMS‐QP2020 NX with autosampler AOC 6000 and a chromatographic column RTx‐5MS (30 m, 0.25 mm i.d., 0.25 µm film thickness) was used. The injection temperature was 210°C (split 1:10), helium was used as carrier gas (pressure 100.0 kPa), and the ionization energy was 70.0 eV by electron ionization. The temperature programming was the following: 40°C–280°C, increasing by 7°C/min, and 280°C for 5 min. The mass spectra were compared to data deposited on library NIST 17; besides, the retention indexes were calculated by injection of alkanes (C9 to C35) and compared to data reported on Adams [37].

### 4.5 FTIR Analysis of HE

The FTIR spectral peaks of HE were recorded in transmittance mode on a Shimadzu IRAffinity‐1 with the following settings: sampling mode KBr; number of scans 500, resolution 4 cm^−1^, and spectral range 400–4000 cm^−1^.

### 4.6 *Culex quinquefasciatus* Larvae

The immature forms of *C. quinquefasciatus* were obtained according to Gerberg [38], with minor modifications in larval rearing. After obtaining the eggs, the larvae were reared in plastic trays (19 cm × 30 cm) containing 0.5 g of rat chow and kept in a natural environment, adapted for the breeding of insects, in the Dona Lindu Center‐West Campus of the Federal University of São João Del‐Rei (CCO/UFSJ).

The experimental design to evaluate the effect of the samples on *C. quinquefasciatus* larvae was developed in accordance with the World Health Organization guideline [39], with minor modifications. Samples (HE, FA30s, FA60s, FAME30s, and FAME60s) were solubilized with 1% DMSO (dimethyl sulfoxide) and tested on *C. quinquefasciatus* at concentrations of 15.62, 31.25, 62.5, 125, and 250 µg/mL. The larvae were placed in contact with 100 mL of each sample and concentration for 24 h. Twenty larvae were placed on each container. After this period, the larvae were transferred to another container containing distilled water. Larvae were counted daily, every 24 h, until death or until complete metamorphosis, and the percentage of mortality was recorded. The experiments were kept inside the Insectarium (CCO/UFSJ), at a temperature of 27°C ± 1°C. All trials, including the positive control group, were carried out in triplicate and repeated three times. The negative control group was performed with 1% DMSO in distilled water, the same solvent used to solubilize the samples. The values of LC_50_ (lethal concentration capable of killing 50% of larvae) and LC_90_ (lethal concentration capable of killing 90% of larvae) were calculated using the probit analysis method [40].

### 4.7 Statistical Analysis

The data were subjected to an analysis of variance (ANOVA). All statistical parameters were calculated using GraphPad Prism 7.0 (San Diego, CA). The criterion for statistical significance was set at *p* < 0.05 by the Tukey test.

## Author Contributions

**Luana Cristina Diniz Santos**: investigation, methodology, formal analyses, writing – review and editing. **Lucas Santos Azevedo**: investigation, methodology, writing – review and editing. **Mairon César Coimbra**: investigation, writing – original draft, writing – review and editing. **Ezequias Pessoa de Siqueira**: methodology. **Stênio Nunes Alves**: conceptualization; funding acquisition. **Ana Hortência Fonsêca Castro**: funding acquisition, supervision, writing – original draft, writing – review and editing. **Luciana Alves Rodrigues dos Santos Lima**: conceptualization, project administration, funding acquisition, data curation; supervision, writing – original draft, writing – review and editing. All authors read and approved the final manuscript.

## Ethics Statement

The work reported in this manuscript complied with all institutional and national policies governing the ethical treatment of the experimental subjects. Our institution does not require ethical approval for studies on invertebrates. This study has access permission to the components of genetic heritage, and it is registered in the SisGen Platform (Register A21AFD9), according to Brazilian Biodiversity Law (13.123/2015).

## Conflicts of Interest

The authors declare no conflicts of interest.

## Declaration of Generative AI in Scientific Writing

The authors declare no use of any AI and AI‐assisted technologies in the article elaboration.

## Supporting information




**Supporting File 1**:  cbdv70301‐sup‐0001‐SuppMat.docx

## Data Availability

Data will be made available on request.
