# Supplementary material for: Larvicidal Activity of Phytochemicals From Handroanthus impetiginosus Seeds Against Culex quinquefasciatus
Source: Chem Biodivers. 2025 Jul 31;22(12):e01126. doi: 10.1002/cbdv.202501126 (PMC12715983; doi:10.1002/cbdv.202501126)

Supporting Information

Figure 1. Total ion chromatogram (TIC) of hexane extract (HE) of *Handroanthus impetiginosus* by GC-MS.

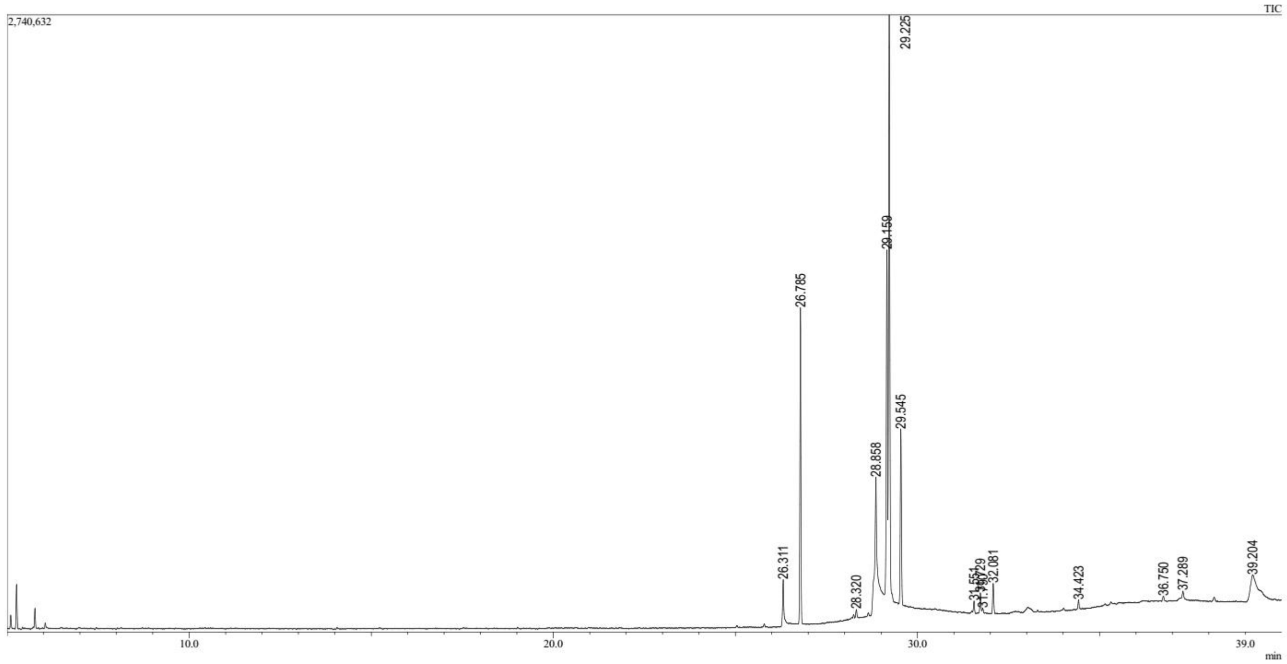

Supplement: Supplementary file 1 — Supporting File 1: cbdv70301‐sup‐0001‐SuppMat.docx [file CBDV-22-e01126-s001.pdf]
